# Supplementary material for: The mouse metallomic landscape of aging and metabolism
Source: Nat Commun. 2022 Feb 1;13:607. doi: 10.1038/s41467-022-28060-x (PMC8807729; doi:10.1038/s41467-022-28060-x)
Supplement: Supplementary file 1 — Supplementary Information [file 41467_2022_28060_MOESM1_ESM.pdf]

The mouse metallomic landscape of aging and metabolism

Supplementary Information

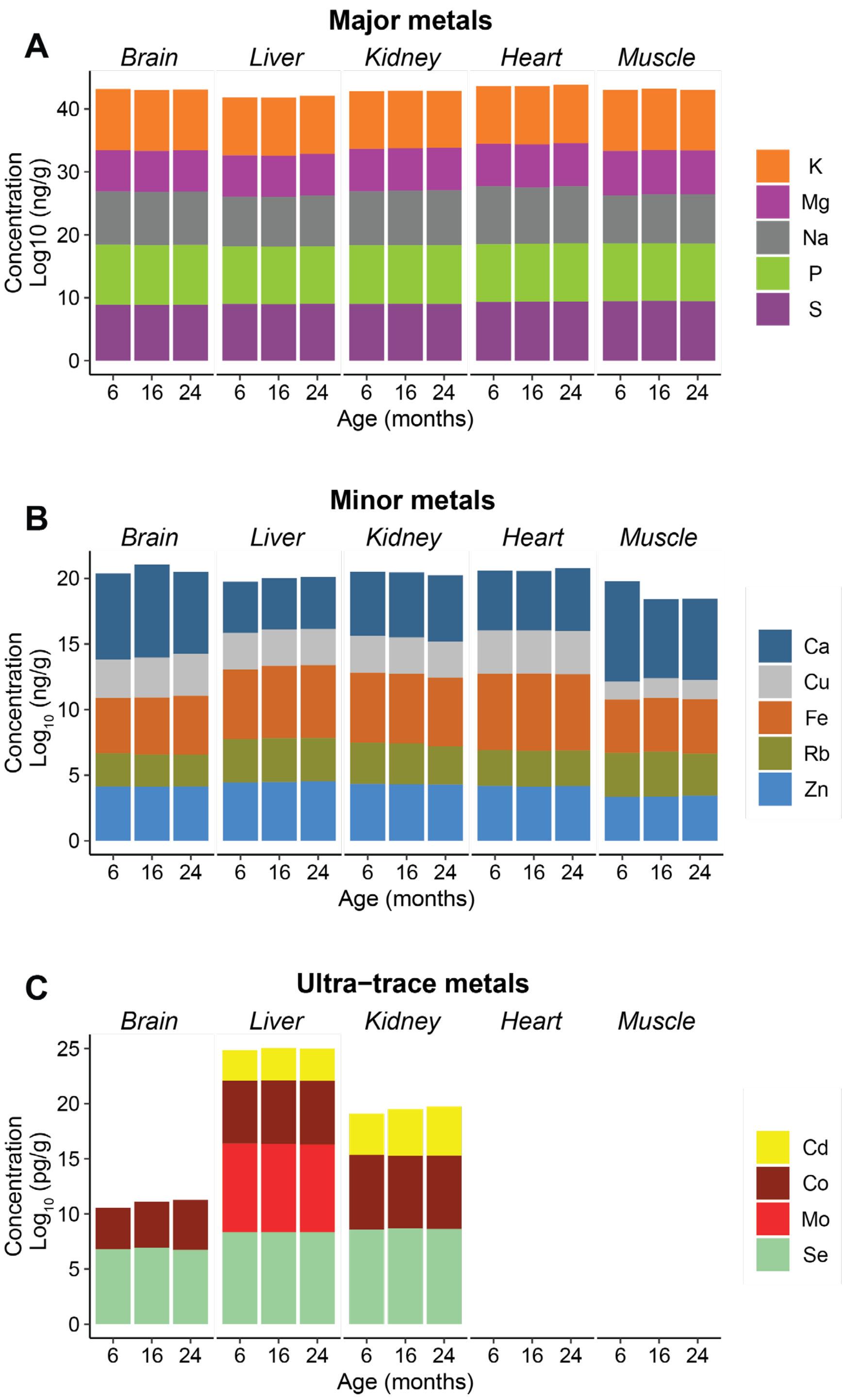

Fig. S1.

Barplots showing the average concentration distribution of (**A**) Major, (**B**) Minor and (**C**) ultra-trace metals in each measured organ and age. Due to the enormous differences in scale between different element, concentrations were converted to log_{10} to enable comparisons

on the same plot. Variations between ages and organs were greater for less abundant metals, and several ultra-trace metals were below the detection limit in heart and muscle which are typically <1 ng/g for the ICP-AES (major metals, **A**) and < 0.1 pg/g for the ICP-MS (minor and ultra-trace metals, **B** and **C**). Source data are provided as a Source Data file.

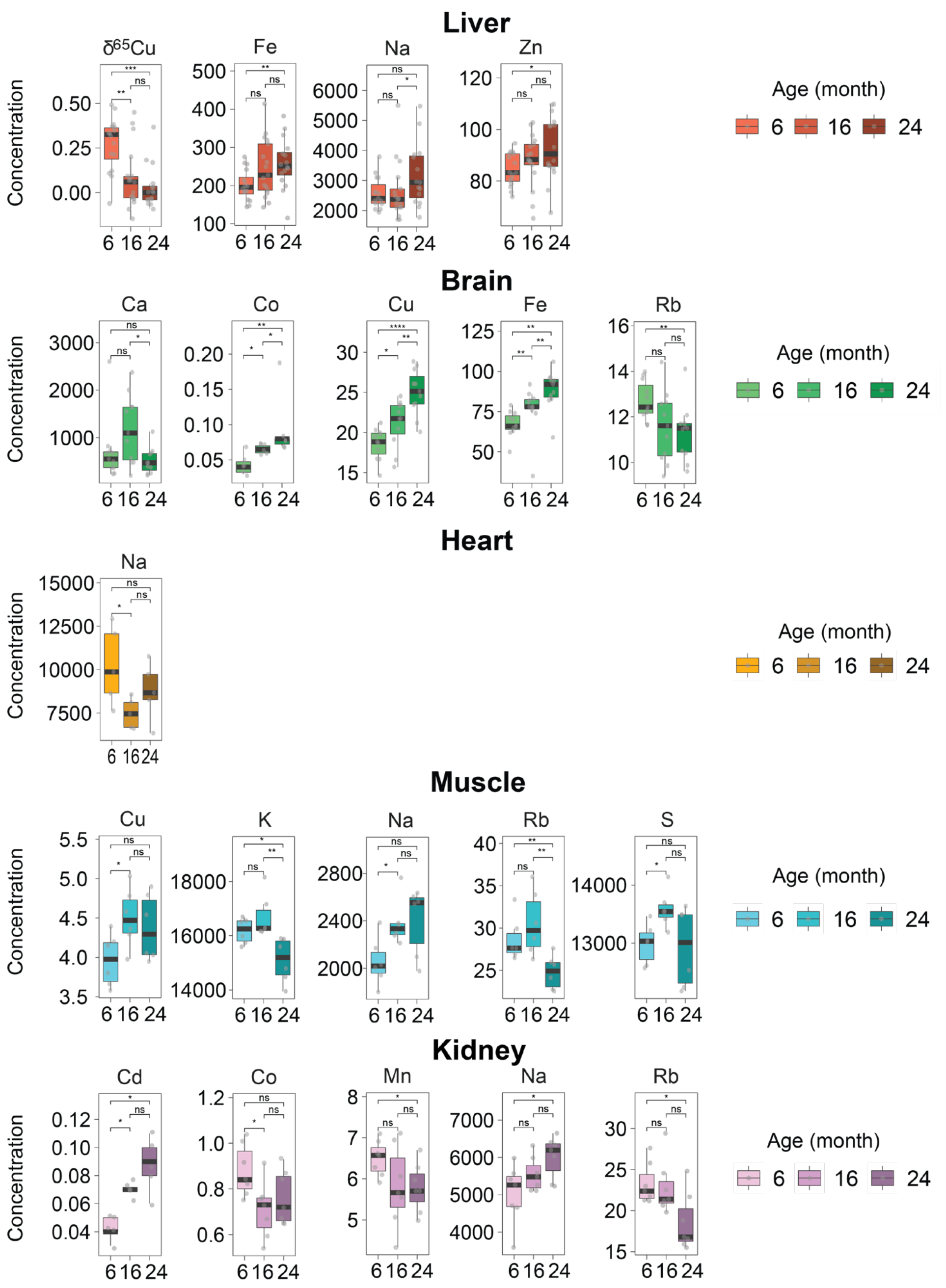

Fig. S2.

Boxplots of the mouse metallome as a function of age and organ. The lower and upper hinges correspond to the first and third quartiles, and center line is the median. The whiskers extend from the hinge to the largest value no further than 1.5 * inter-quartile range. Only metals with significant (Adj. *P*-value < 0.05) variations are shown. Cu and Fe significantly

increase with age in brain, a factor that may relate to cognitive decline, and Cd significantly increase with age in brain. Of note, is the decrease between 6 and 24 mo for Rb and K in several organs, suggesting evidence of increasing K dysregulation during aging. ns non-significant, * Adj.$P < 0.05$, ** Adj.$P < 0.01$, *** Adj.$P < 0.001$, **** Adj.$P < 10^{-4}$, FDR-corrected (limma) moderated t-test, two-sided. Source data are available on table S1.

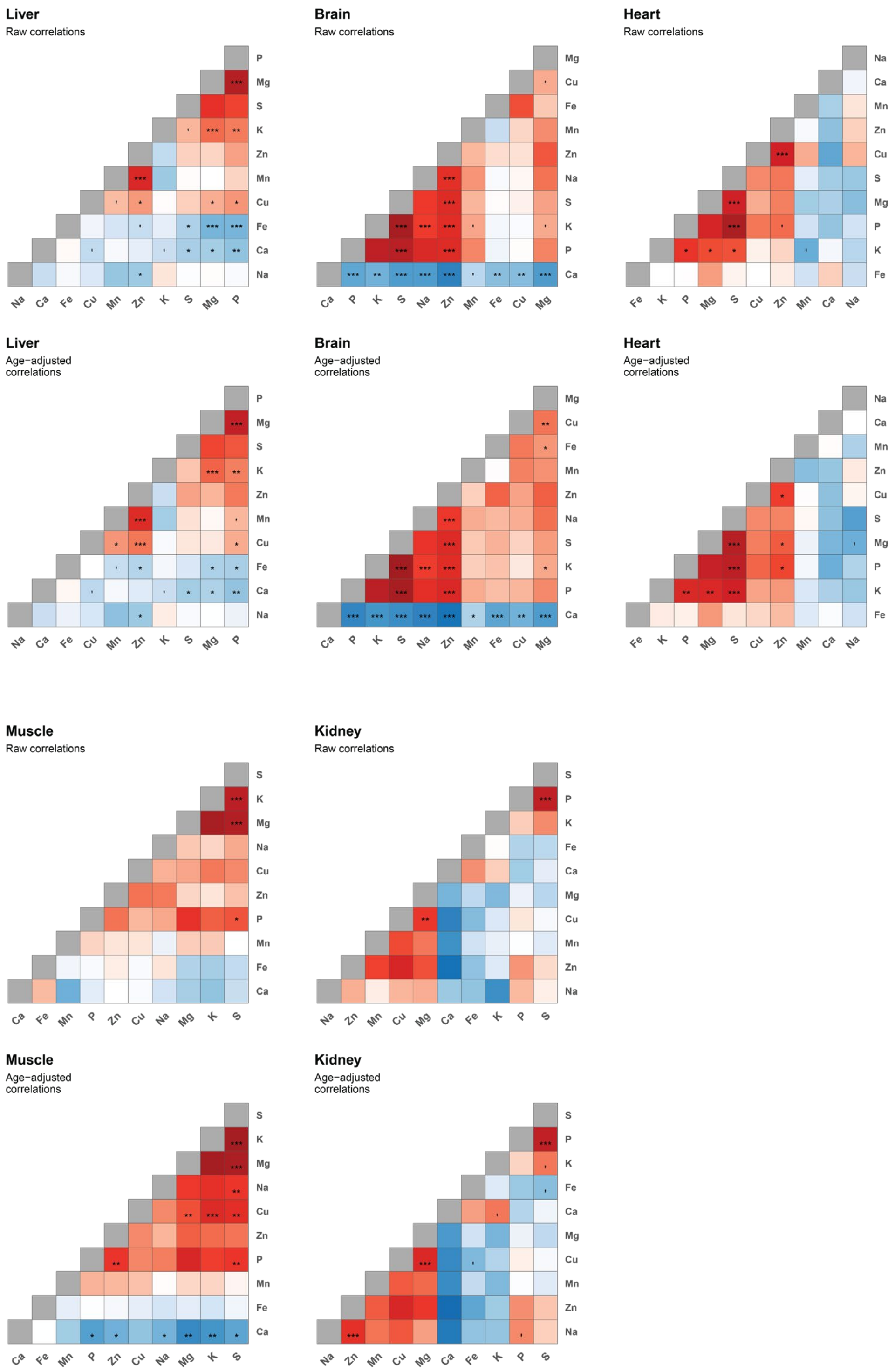

Fig. S3.

Heatmaps of Spearman Rho coefficients of the mouse metallome for each organ. For a given organ, heatmaps are provided for raw concentrations in the upper panel and for age-corrected concentrations in the lower panel. Metals are ordered by hierarchical clustering, based on the age-adjusted data in each organ. Generally, age correction does not affect

correlations between elements, notably the positive S vs P and negative Ca vs Mg correlations. * Adj.$P < 0.05$, ** Adj.$P < 0.01$, *** Adj.$P < 0.001$, FDR-corrected limma linear regression test, with age as a co-variate in age-corrected panels. Source data are provided as a Source Data file.

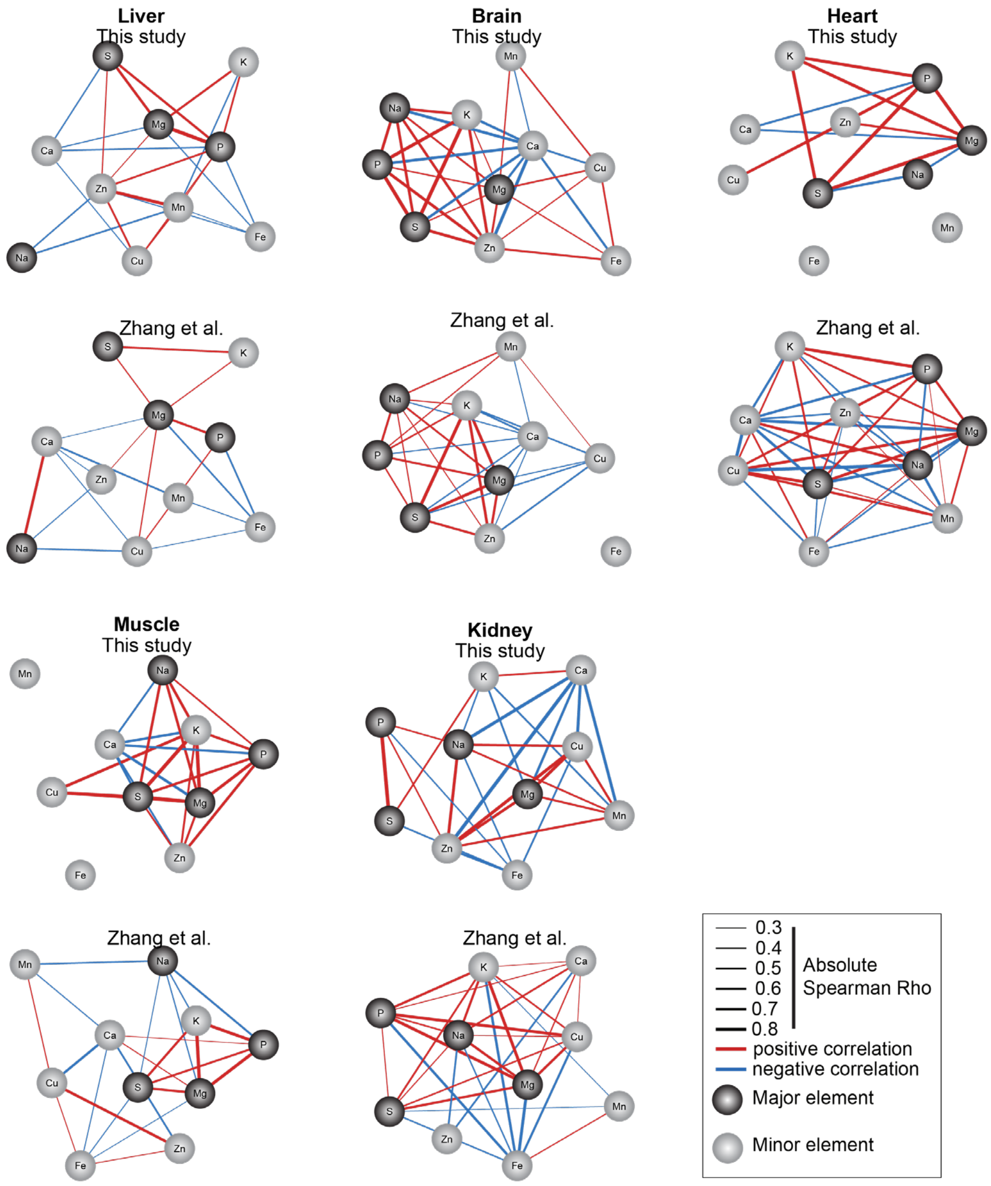

Fig. S4.

Networks of Spearman Rho correlations for the mouse metallome in different organs, showing conservation between data from this study (upper network) and from Zhang *et al. (10)* (lower network). Only significant (Adj.P < 0.05) correlations are considered, and concentrations are age-corrected. A more systematic comparison between the correlation coefficients of both networks can be found on Fig 2B. The networks in liver, brain and heart are very similar, while muscle shows intermediate, and kidney poor, conservation. Source data are provided as a Source Data file.

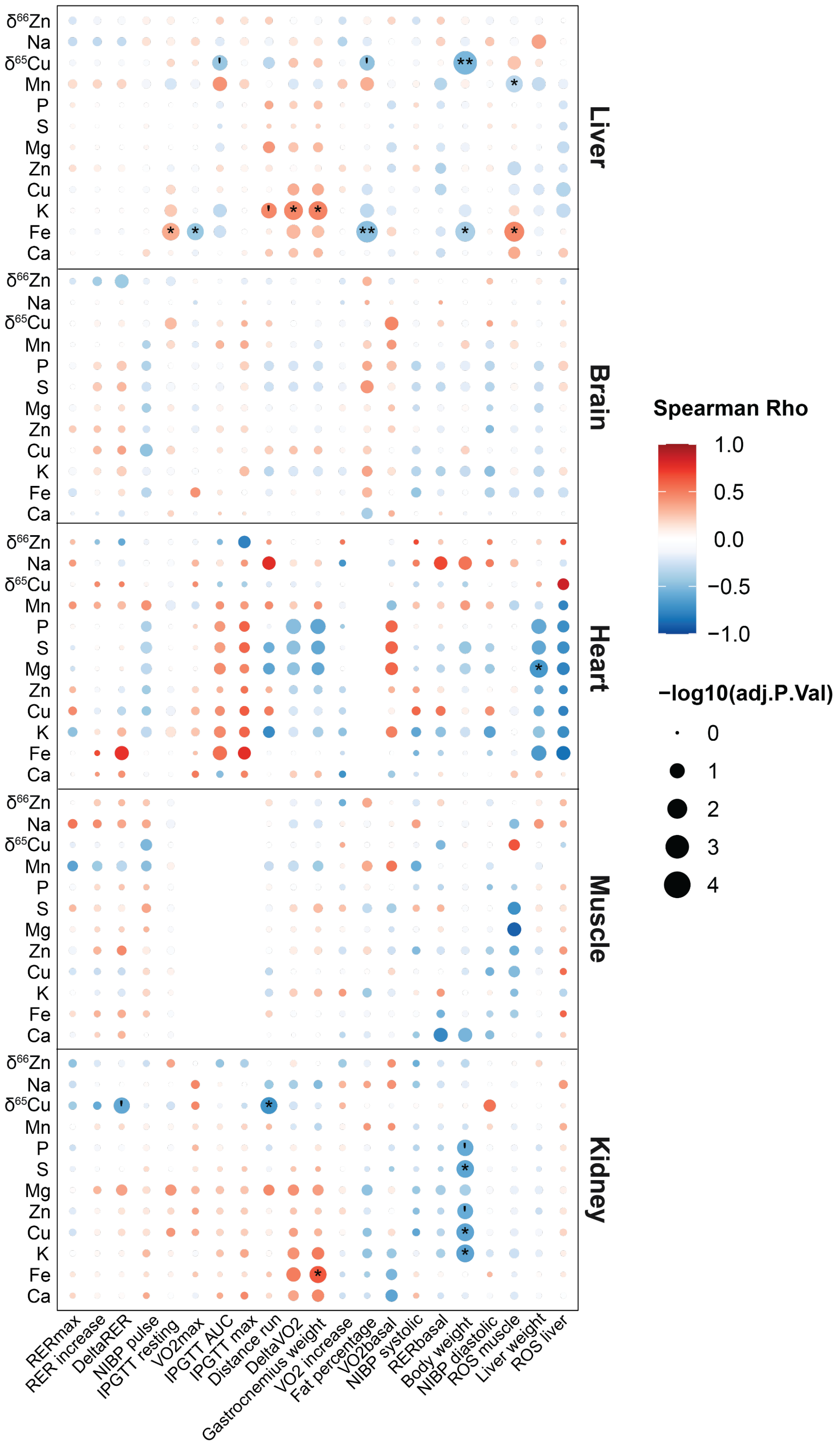

δ66Zn
Na
δ65Cu
Mn
P
S
Mg
Zn
Cu
K
Fe
Ca
Liver
Brain
Heart
Muscle
Kidney
RERmax
RER increase
DeltaRER
NIBP pulse
IPGTT resting
VO2max
IPGTT AUC
IPGTT max
Distance run
DeltaVO2
Gastrocnemius weight
VO2 increase
Fat percentage
VO2basal
NIBP systolic
RERbasal
Body weight
NIBP diastolic
ROS muscle
Liver weight
ROS liver
Spearman Rho
1.0
0.5
0.0
−0.5
−1.0
−log10(adj.P.Val)
0
1
2
3
4

Fig. S5. Heatmaps of Spearman Rho coefficients between the mouse metallome of each organ, and the phenotypes. The kidney and liver are the only organs with significant correlations (and a single one for the heart), which is consistent with the fact that all the measured phenotypic traits are metabolic in essence and are therefore more likely to involve the liver or kidney. ***Adj.P < 0.001, **Adj.P < 0.01, *Adj.P < 0.05, Adj.P < 0.1, FDR-corrected, two sided, (limma) moderated t-test with age as a covariate. Source data are provided as a Source Data file.

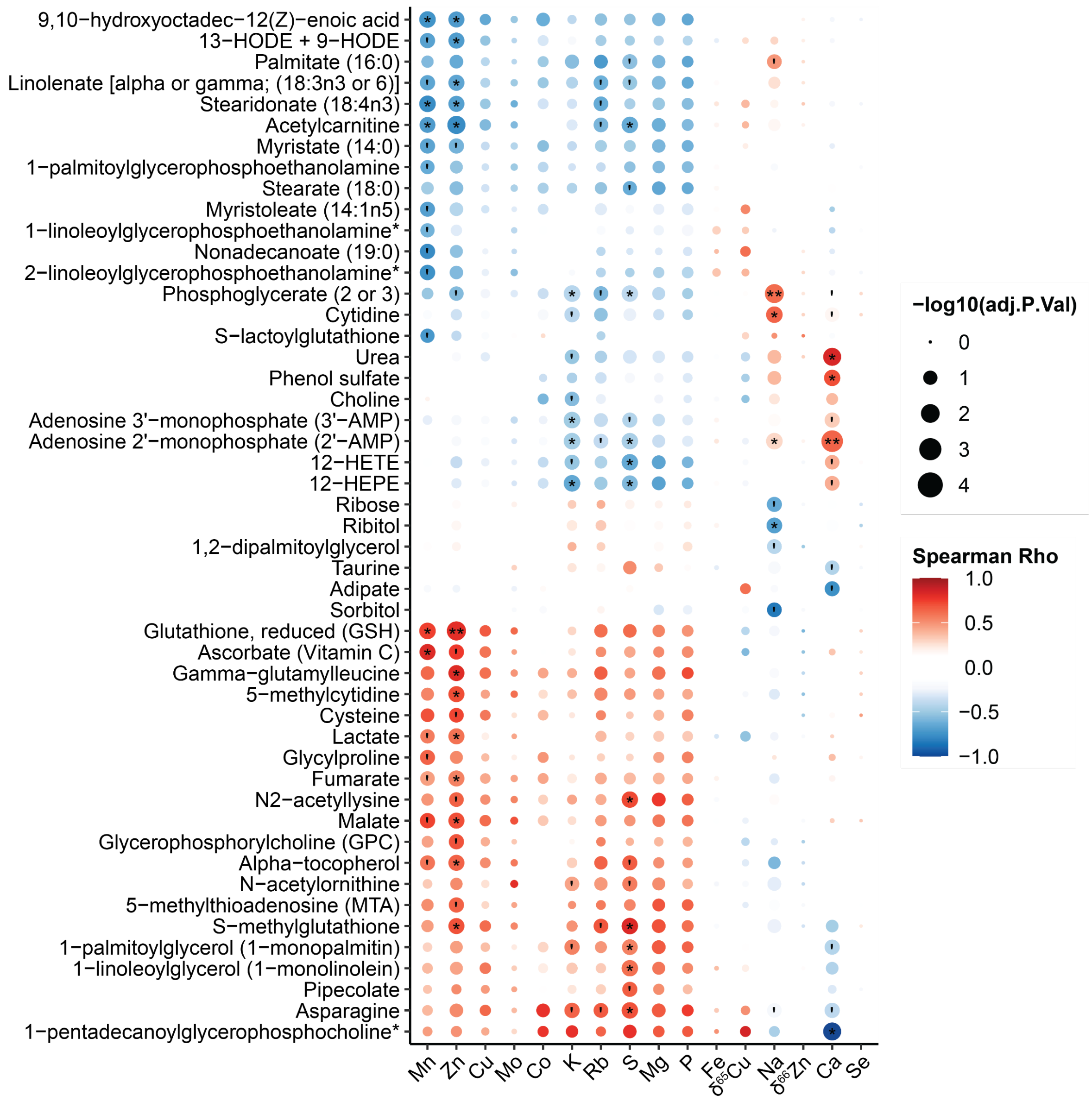

Fig. S6. Heatmaps of Spearman Rho coefficients between the liver metallome and the metabolome. Only metabolites with at least one correlation at Adj.P < 0.1 are displayed. Except for ultra-trace metals and δ^{66}Zn, most of the metals exhibit numerous associations, of which the strongest mostly involve Zn and S. ***Adj.P < 0.001, **Adj.P < 0.01, *Adj.P < 0.05, Adj.P < 0.1 FDR-corrected, two sided, (limma) moderated t-test with age as a covariate. Source data are provided as a Source Data file.

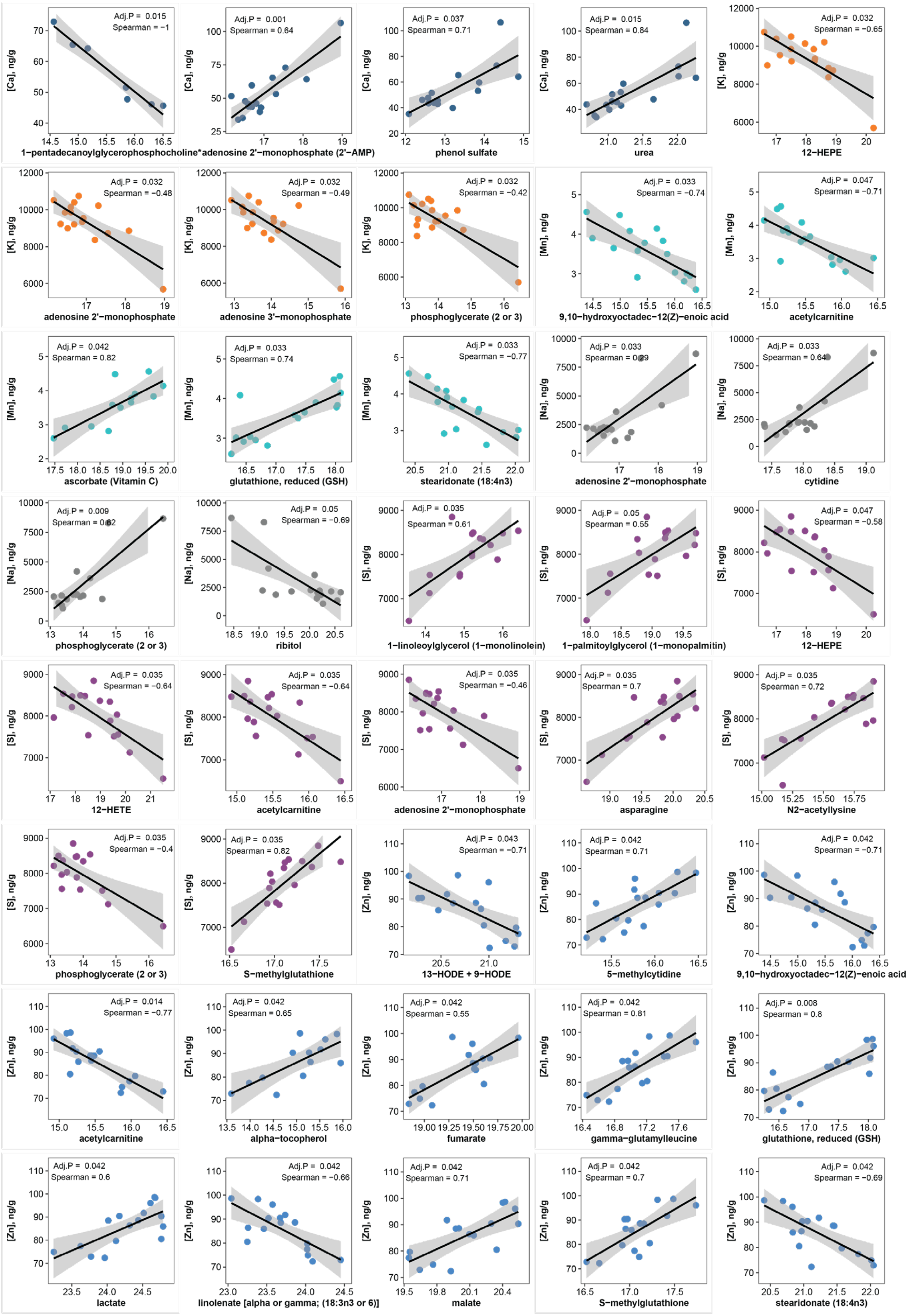

Fig. S7.

Scatterplots between the mouse metallome and metabolome. Only significant correlations (adj.*P* < 0.05) are shown. The black line and grey error band represent a linear regression

and its 95% confidence interval, respectively. FDR-corrected, two sided, (limma) moderated t-test with age as a covariate. Source data are provided as a Source Data file.

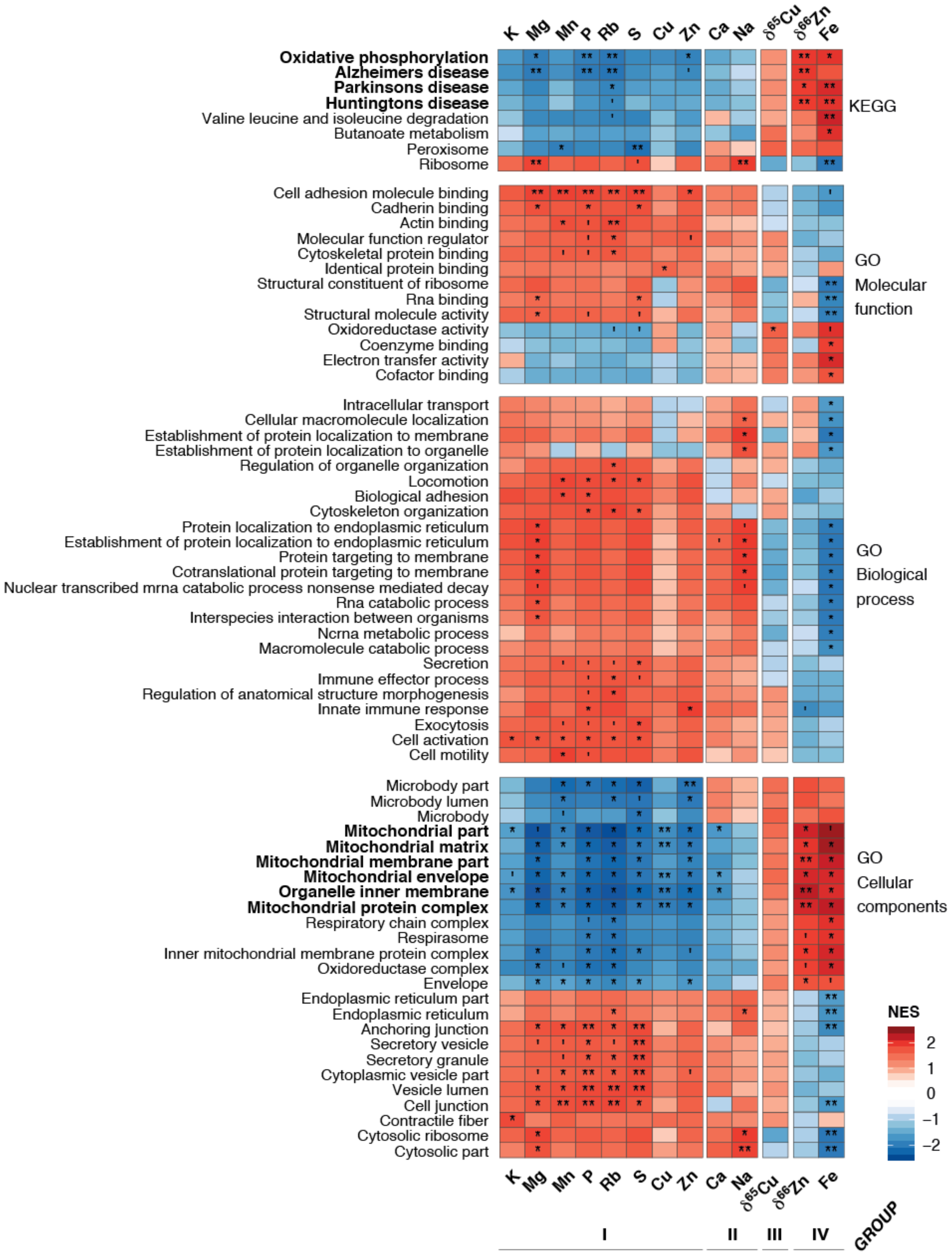

Fig. S8. Proteins were sorted on their correlation with individual metals in the liver, then

gene-set enrichment analysis (GSEA) was performed. For each metal, the top five gene sets are selected in each category and included in the heatmap. Gene sets discussed in the main text are highlighted in bold. FDR-corrected pvalues: ' Adj.P < 0.1, * Adj.P < 0.05, ** Adj.P < 0.01, ** Adj.P < 0.001, *** Adj. < 10^{-4}. Source data are provided as a Source Data file.

	This study		Reference values	
	OEP	SRM-1577c	OEP	SRM-1577c
**Mn**	n/a	9.9±0.2 (4)	n/a	10.5±0.5**
**Fe**	20.1±4.6	191±5 (4)	19.5±3.6 (15)*	198±1**
**Co**	0.17±0.03 (3)	0.29±0.01 (4)	0.17±0.04 (15)*	0.30±0.02**
**Cu**	10.7±1.2 (3)	270±5 (4)	10.5±1.4 (15)*	273±5**
**Zn**	7.3±1.0 (3)	177±4 (4)	7.2±1.2 (15)*	181±1**
**Se**	1.33±0.22 (3)	1.67±0.19 (4)	1.48±0.77(15)*	2.03±0.04**
**Rb**	1.10±0.29 (3)	31.8±0.6 (4)	1.03±0.18(15)*	35.3±1.1**
**Mo**	2.70±0.66 (3)	3.7±0.2 (4)	2.61±0.39(15)*	3.3±0.1**
**Cd**	n/a	0.08±0.02 (4)	n/a	0.10±0.01 **
**Ca**	900±171 (3)	134±6 (4)	870±144(15)*	131±10**
**K**	2079±395 (3)	10355±261 (4)	1989±405(15)*	10230±640**
**Na**	36667±7751 (3)	1985±58 (4)	34494±8248(15)*	2033±64**
**P**	1085±101 (3)	11070±249 (4)	1068±152(15)*	11750±270**
**S**	10156±1734 (3)	7162±178 (4)	9792±1477(15)*	7490±340**
**Mg**	257±38 (3)	587±18 (4)	272±66(15)*	620±42**
**δ^{65}Cu**	-1.10±0.15 (3)	0.43±0.04 (4)	-1.14±0.13 (35)*	0.37±0.14 (45)*
**δ^{66}Zn**	0.76±0.08 (3)	-0.18±0.05 (4)	0.73±0.09 (35)*	-0.19±0.06 (45)*

Table S1.
Element concentrations (ppm) and Cu-Zn isotopic compositions (‰) of in-house and certified reference standards. * long-term in-lab average. ** reference value from National Institute of Standards and Technology.
