# Supplementary material for: The mouse metallomic landscape of aging and metabolism
Source: Nat Commun. 2022 Feb 1;13:607. doi: 10.1038/s41467-022-28060-x (PMC8807729; doi:10.1038/s41467-022-28060-x)
Supplement: Supplementary file 3 — Description of Additional Supplementary Files [file 41467_2022_28060_MOESM3_ESM.docx]

**Description of Additional Supplementary Files**

**File Name:** Supplementary Data 1

**Description:** Element concentrations (ppm) and Cu-Zn isotopic compositions of mouse organs.
