# Supplementary material for: The mouse metallomic landscape of aging and metabolism
Source: Nat Commun. 2022 Feb 1;13:607. doi: 10.1038/s41467-022-28060-x (PMC8807729; doi:10.1038/s41467-022-28060-x)
Supplement: Supplementary file 5 — Reporting Summary [file 41467_2022_28060_MOESM5_ESM.pdf]

nature research

Corresponding author(s): Vincent Balter

Last updated by author(s): Sep 13, 2021

Reporting Summary

Nature Research wishes to improve the reproducibility of the work that we publish. This form provides structure for consistency and transparency in reporting. For further information on Nature Research policies, see our Editorial Policies and the Editorial Policy Checklist.

Statistics

For all statistical analyses, confirm that the following items are present in the figure legend, table legend, main text, or Methods section.

n/a	Confirmed	
[ ]	[x]	The exact sample size (*n*) for each experimental group/condition, given as a discrete number and unit of measurement
[ ]	[x]	A statement on whether measurements were taken from distinct samples or whether the same sample was measured repeatedly
[ ]	[x]	The statistical test(s) used AND whether they are one- or two-sided<br>*Only common tests should be described solely by name; describe more complex techniques in the Methods section.*
[ ]	[x]	A description of all covariates tested
[ ]	[x]	A description of any assumptions or corrections, such as tests of normality and adjustment for multiple comparisons
[ ]	[x]	A full description of the statistical parameters including central tendency (e.g. means) or other basic estimates (e.g. regression coefficient) AND variation (e.g. standard deviation) or associated estimates of uncertainty (e.g. confidence intervals)
[ ]	[x]	For null hypothesis testing, the test statistic (e.g. *F*, *t*, *r*) with confidence intervals, effect sizes, degrees of freedom and *P* value noted<br>*Give P values as exact values whenever suitable.*
[x]	[ ]	For Bayesian analysis, information on the choice of priors and Markov chain Monte Carlo settings
[ ]	[x]	For hierarchical and complex designs, identification of the appropriate level for tests and full reporting of outcomes
[ ]	[x]	Estimates of effect sizes (e.g. Cohen's *d*, Pearson's *r*), indicating how they were calculated

Our web collection on statistics for biologists contains articles on many of the points above.

Software and code

Policy information about availability of computer code

Data collection: Major element concentrations were measured using iTEVA 2.2.0.51, trace element concentrations using Qtegra 2.10.4345 and isotope compositions using Nu Plasma 1.4.1006.

Data analysis: Data analysis was performed in R software version 3.5.2. The following open-source packages were used in analysis and figure generation: MsqRobSum version 0.9: proteomics ; limma version 3.38.3: differential analyzes and parallel linear regressions; ClusterProfiler version 3.10.1: Gene and Metabolite set enrichment analyzes; ggplot2 version 3.3.2: figure generation; igraph version 1.2.4.1, ggraph version 1.0.2: network visualizations. All code used in the paper is available on request .

For manuscripts utilizing custom algorithms or software that are central to the research but not yet described in published literature, software must be made available to editors and reviewers. We strongly encourage code deposition in a community repository (e.g. GitHub). See the Nature Research guidelines for submitting code & software for further information.

Data

Policy information about availability of data

All manuscripts must include a data availability statement. This statement should provide the following information, where applicable:

- Accession codes, unique identifiers, or web links for publicly available datasets
- A list of figures that have associated raw data
- A description of any restrictions on data availability

Raw metallomic data are included in this study Supplemetary data 1. Proteomics have been submitted to the PRIDE repository under the identifier PXD011142 (https://www.ebi.ac.uk/pride/archive?keyword=PXD011142). Metabolomics data have been previously published and are available in ref 41 (https://www.ncbi.nlm.nih.gov/pmc/articles/PMC3216615/).

Field-specific reporting

Please select the one below that is the best fit for your research. If you are not sure, read the appropriate sections before making your selection.

☒ Life sciences ☐ Behavioural & social sciences ☐ Ecological, evolutionary & environmental sciences

For a reference copy of the document with all sections, see nature.com/documents/nr-reporting-summary-flat.pdf

Life sciences study design

All studies must disclose on these points even when the disclosure is negative.

Sample size	Given the novelty of metal measurements in mammalian organs, no prior data was available to estimate variability and therefore sample size. Therefore, we chose a conservative sample size of 16 mice per age group for metallomics. Metabolomic (n=8) and proteomic (n=4) analyzes were already performed and published prior to our study, and therefore sample size could not be changed.
Data exclusions	For metallomics, outlier measurements were performed through the R package 'outliers' using the 'normal' method (differences between each value and the mean divided by standard deviation) . Less than 5% of measurements were removed this way. No samples were excluded in other 'omic' layers.
Replication	The paper specifically examines the reproducibility of the organ distribution, age effects and correlations between metals by comparing with a recent study (see figure 2 and paragraph "The metallome fingerprint is highly conserved across different studies")
Randomization	Allocation of animals to the three age groups was performed randomly at weaning.
Blinding	All mice were assigned a non-explicit ID , which was used for all measurements. However the metadata relative to these IDs was available to most collaborators, so there was no specific blinding. Nevertheless all analysis was processed directly from raw mass spectrometry data, which should not present subjective bias.

Reporting for specific materials, systems and methods

We require information from authors about some types of materials, experimental systems and methods used in many studies. Here, indicate whether each material, system or method listed is relevant to your study. If you are not sure if a list item applies to your research, read the appropriate section before selecting a response.

Materials & experimental systems

n/a	Involved in the study	
☒	☐	Antibodies
☒	☐	Eukaryotic cell lines
☒	☐	Palaeontology and archaeology
☐	☒	Animals and other organisms
☒	☐	Human research participants
☒	☐	Clinical data
☒	☐	Dual use research of concern

Methods

n/a	Involved in the study	
☒	☐	ChIP-seq
☒	☐	Flow cytometry
☒	☐	MRI-based neuroimaging

Animals and other organisms

Policy information about studies involving animals; ARRIVE guidelines recommended for reporting animal research

Laboratory animals	All mice were male C57BL/6J mice, purchased from Janvier, St Berthevin, France. Animals were were sacrificed at three timepoints at 6, 16 or 24 months of age.
Wild animals	This study did not involve wild animals.
Field-collected samples	This study did not involve samples collected in the field.
Ethics oversight	All animal experiments were performed according to Swiss ethical guidelines and approved by the local animal experimentation committee of the Canton de Vaud under license 2172

Note that full information on the approval of the study protocol must also be provided in the manuscript.
